# A Common Cause of ADHD and Bipolar Disorder (BD)

**DOI:** 10.1111/bdi.70057

**Published:** 2025-09-01

**Authors:** Rabinovitch Avinoam, Rabinovitch Revital, Braunstein Doron, Smolik Ella, Biton Yaacov

**Affiliations:** ^1^ Physics Department Ben‐Gurion University Beer‐Sheva Israel; ^2^ Makif YudAlef Rishon Lezion Israel; ^3^ Physics Deptartment Sami Shamoon College of Engineering Beer‐Sheva Israel

**Keywords:** ADHD, bipolar, disorder

## Abstract

**Objectives:**

Bipolar and ADHD are two diseases that exhibit similar traits but at different levels of severity. Understanding this behavior can help treatment of these disorders.

**Method:**

We hypothesize that there is a common partial genetic cause for both attention‐deficit/hyperactivity disorder (ADHD) and bipolar disorders (BD), which is the reversal function of the dopamine neurotransporter DAT gate (see Figure 1) into an efflux mode. We analyze the dopamine chemistry in the neuron synapse under this assumption.

**Results:**

The difference between ADHD and BD phenotypes is due either to the dopamine receptor (D2R) operation or to the threshold levels of the dopamine homeostatic control and the intensity of its operation.

**Significance:**

The common cause of the two disorders could explain the frequent phenomenon of ADHD symptoms preceding BD beginning and the high comorbidity between these disorders.

## 1 Introduction

Bipolar disorder (BD) is a mental health disorder that causes periods of ups and downs in a person's mood, with possible “euthymia” or normal behavior periods, interspersed. The “up” times can be severe and are referred to as mania, or less severe episodes are referred to as hypomania [1]. During the “down” (depression) times, the person has a negative outlook on life, and the risk of suicide is increased [1]. This disorder occurs in ~1% of the population worldwide and causes morbidity and possible increased mortality.

ADHD is an abundant neurodevelopmental disorders affecting ~370 million adults worldwide and ~20 million individuals (5.2% of the adult population) in the USA [2]. According to the DSM‐5 Diagnostic Criteria [3], there are three types of syndromes of ADHD sufferers: predominantly Inattentive Type (PIT), predominantly Hyperactive/Impulsive Type (PHT), and a Combined Type.

For many years, the changing concentrations of the organic chemical Dopamine (DA) have been recognized as a possible cause of BD [4, 5] and of ADHD [6]. DA has a major influence on a person's moods; for example, it is well known that during the manic episodes of BD, brain DA concentrations are elevated, while during the depression episodes, they are reduced [7]. Moreover, medications blocking dopamine receptors are used to treat manic symptoms.

## 2 The Hypothesis

**FIGURE 1:**
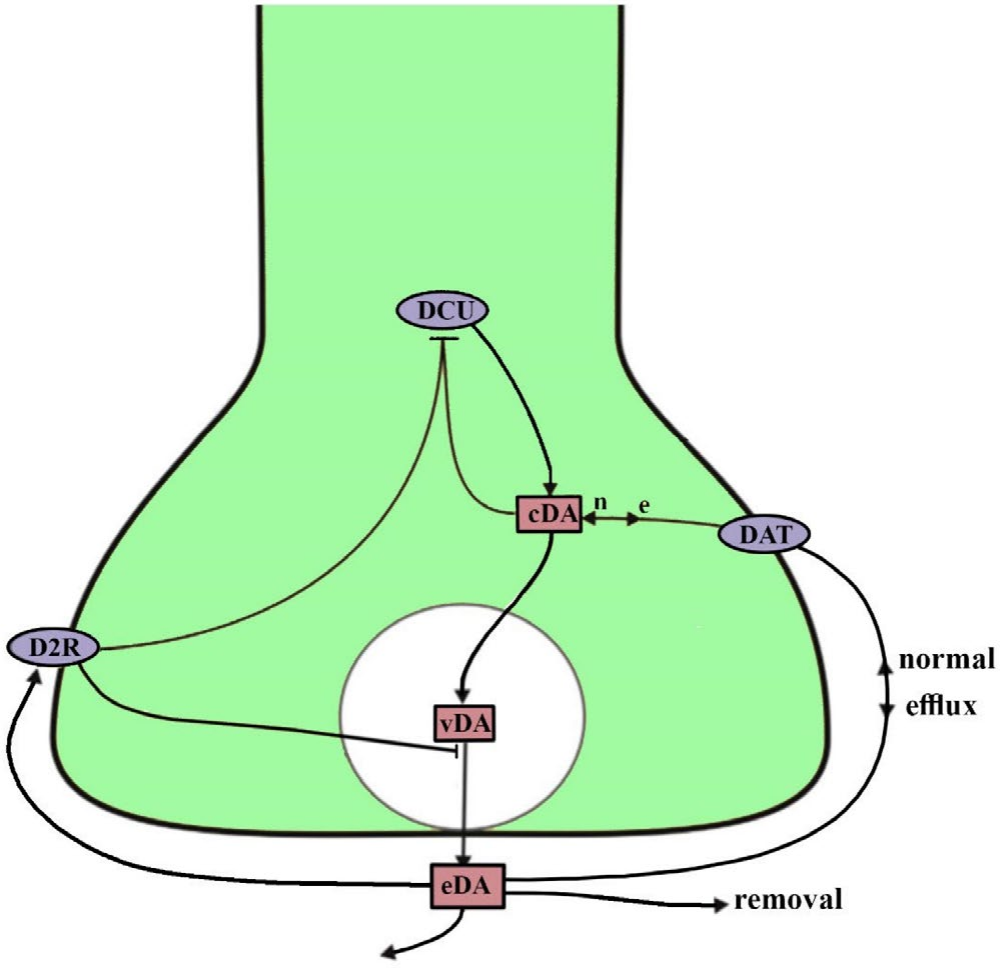
Main dopamine (DA) processes, in and out of a dopaminergic synaptic terminal. DA is chemically created in the DCU section and can emerge from the vesicle (the white sphere) on a cue from the brain system. The extracellular dopamine (eDA) can alter a person's mood and behavior. Normally, part of it is reabsorbed by the synapse through the DAT operation, and another part of DA is removed directly. Excess extracellular (eDA) concentrations open a D2 receptor operation limiting both DA production and vesicular DA exit. Adapted from [8]. cDA, cytoplasm dopamine; DA, dopamine; DCU, dopamine creating unit; eDA, extracellular space dopamine; e, efflux; n‐normal; vDA, vesicular dopamine.

**FIGURE 2:**
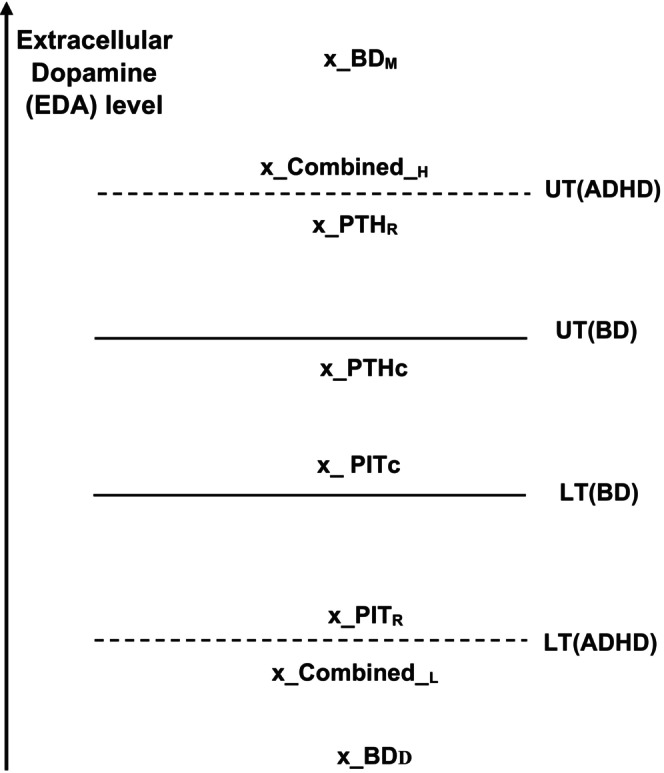
A schematic chart of possible DAT regulation upper and lower thresholds for ADHD and bipolar disorders for regular and comorbid patients. Dopamine levels are shown relative to these thresholds for stationary states of all types of these disorders. BD_M_ and BD_D_, possible dopamine levels of the manic and depressive bipolar stages; Combined_H_ and Combined_L_, possible dopamine levels of the high and low stages of the combined ADHD type; PHT_C_ and PIT_C_, possible dopamine levels of the comorbid stages of these types; PHT_R_ and PIT_R_, possible dopamine levels of regular (not comorbid with the bipolar disorder) stages of the predominantly Inattentive Type (PIT) and the predominantly Hyperactive/Impulsive Type (PHT) types.

1. The regular function of the DAT neuro‐transporter gates is to transfer dopamine molecules from the extracellular space (ES) into the neural synapses from which they have previously emerged (Figure 1). However, under substance misuse, this function reverses. Instead of “reuptake” where the dopamine molecules are transferred from the ES into the synapse, it drives them out of the synapses and into the ES. This procedure is called “efflux.” The DAT functionality direction also reverses in pathological situations of genetic mutations. This reversal is called anomalous efflux of dopamine (ADE). Several mutations are known to reverse the DAT function, for example, the hDAT coding A559V variant [9, 10]. This feature was observed in behavioral phenotypes of knock‐in animal models ([11]; see also the review and model observations of Mayer et al., 2023, [12]). Other mutations exhibiting this phenomenon are the T356M and the D421N variants [13]. This pathological DAT operation [13] is also notorious for supporting a significant impact of ADE in vivo [14]. We believe that mutations leading to DA efflux [9, 15, 16, 17], have the potential to cause both bipolar disorder and ADHD.
2. Once ADE occurs in the brain, the brain detects a surplus of extracellular dopamine (eDA) concentration, and the D2R control mechanism (Figure 1) starts to operate [18]. It inhibits the manufacture of DA in the relevant neuron, leading to a lower number of DA molecules leaving its synapse, both from vesicles (one vesicle is simulated in Figure 1 by a white sphere) and the DAT efflux. This process can cause a drop in eDA to even below‐normal levels. Such low levels are detected in the brains of some ADHD sufferers [19].
3. The *normal* dopamine (DA) balance in the brain is partly maintained by a homeostasis system controlling DAT and guiding the intensity and duration of DA presence, either by phosphorylation [20] or by Kinase‐dependent [21] regulation. If the extracellular dopamine, eDA, concentration increases above a certain threshold (upper threshold, UT), this system upsurges the DAT operation, bringing a current of DA back into the neural synapses (see Figure 1) and thus lowering the eDA level. On the other hand, if the eDA level decreases below a different threshold (lower threshold, LT), the system induces the DAT neuro transporters to close, leading to a gradual eDA increase. This control is carried out by proteins linked to DAT that regulate its distribution, functional properties, and modulation by psychostimulants [22, 23].
  An unwanted positive feedback process occurs under a *reversed* (ADE) DAT operation: If the eDA level increases above the UT, the control system would still drive the DAT gates to open, leading to even more DA efflux from the synapses by the transformed DAT openings, causing an even higher eDA level, etc. An inverse positive feedback procedure would occur for situations in which the eDA level drops below the LT. There, closing the DAT gates would eventually lead to lower and lower eDA levels.
4. We assume that in both ADHD and bipolar disorders (BD), the mechanism of the DAT gates is partially or fully genetically damaged, in that their function is reversed; instead of returning the DA into the neuron, they operate to drive DA out of it (abnormal dopamine efflux, ADE). Incidentally, this common cause of the two disorders could explain the well‐known fact that ADHD symptoms frequently precede BD inception [24, 25] which starts at a later stage in life, and the high comorbidity between these disorders [26].
  The difference between these disorders can partly be in the high (UT) and low (LT) levels of eDA, which induce the homeostasis apparatus to step in. We contend that these levels are much further apart in some ADHD types than in the BD syndrome (see Figure 2). Thus, while people with ADHD can remain euthymic with a broad range of eDA, it is relatively easy for a BD patient to step out of this state into either a manic or a depressive one under the operation of the positive feedback system. See also item 6 below.
5. The psychostimulant drugs, for example, Methylphenidate (Ritalin) and Amphetamine, are used as therapies for ADHD. Their influence is carried out by directly inhibiting dopamine transporters (DAT). This fact supports the assumption of “efflux” (ADE) as the cause of the disorder since restricting DAT reduces the efflux and also the reversed function of the homeostatic feedback control of this gate, and thus resolves the “paradox” of using “stimulants” to treat ADHD [27].
6. As mentioned above, according to the DSM‐5 Diagnostic Criteria, there are three types of syndromes of ADHD sufferers: predominantly Inattentive Type (PIT), predominantly Hyperactive/Impulsive Type (PHT), and a Combined Type. We assume that, like in BD, in people with ADHD, the DAT gates operate (partly or fully) under the ADE mode, but either the $\left(\mathrm{UT}-\mathrm{LT}\right)$‐range or the DAT homeostasis feedback is different. We reckon that in the first two ADHD types (PIT and PHT), the range mentioned above is much broader than BD's, letting ADHD of these types stay “euthymic”, at higher (PHT) or lower (PIT) dopamine levels, without resorting to the homeostasis feedback machinery. These levels can be obtained “merely” by the D2R controlling system regulation intensity. Thus, suppose enough membrane‐associated DAT gates are operating in the efflux mode. This should lead to a high level of eDA. The D2R can regulate this level in varying intensities, leading to a steady state of a low or high eDA level, and the first two syndromes (Figure 2).
  On the other hand, the Combined syndrome is possibly a mild version of BD where the range $\left(\mathrm{UT}-\mathrm{LT}\right)$ *is* exceeded, and the homeostasis regulation of DAT is initiated. However, the homeostasis positive feedback loop is weak, and the two final eDA levels are not as extreme as in the BD case (Figure 2).
  We, therefore, assume that the comorbidity of ADHD with BD can be more pronounced in the Combined Type of the former disorder.

Figure 2 schematically depicts our hypothesis. The difference between the dopamine levels of the ADHD types is emphasized. Thus, the dopamine levels of people, while being in the comorbid BD‐ADHD stage, are still in the euthymic BD range. PHT and PIT patients, not in comorbidity with BD, can have a larger dopamine range. Dopamine levels of people of the combined type can reside outside of *their* $\left(\mathrm{UT}-\mathrm{LT}\right)$‐range (not necessarily the same as the range of the other types, which is the one shown here). Still, all ADHD dopamine levels. including those of the combined type, are assumed to be less extreme than the BD's. Therefore, ADHD patients show much milder symptoms than BD patients.

In conclusion, our assumption that bipolar and ADHD disorders have a common cause, the reversal function of the DAT gate, could explain their comorbidity and help their treatments. Evidently, being a novel idea, to be valid, our hypothesis should be experimentally verified. We urge experimentalists to investigate this possibility by comparing the DAT function in people with and without these disorders.

## Conflicts of Interest

The authors declare no conflicts of interest.

## Data Availability

The data that support the findings of this study are available from the corresponding author upon reasonable request.
